# The Royal Infirmary and Our Prisoners in Germany: An Example Which Might Be Followed

**Published:** 1917-06-30

**Authors:** 


*To the Editor of* The Hospital.

### The Royal Infirmary and our Prisoners in Germany: An Example which might be Followed.

*To the Editor of* The Hospital.

"Take care of the pence, and the pounds will take care of themselves"; so runs the adage, and the war has proved its truth. Almost every week enormous sums are raised for war charities by means of Flag Days, and we are at last beginning to realise that our smallest savings and humblest gifts can promote the great cause we all have at heart. In general hospitals, except where there are military patients, though the work is of national importance, it is of a purely civil character, and the nurses feel keenly that they cannot engage in some form of "war work." Their profession is closely linked with the Army, and many of them count the days till they may join one of the Services; but they have little money and less time, and their desire to "help the war" remains in the main unsatisfied. In the Royal Infirmary of Edinburgh it has found expression in a sustained effort to aid our prisoners of war—four of whom are dependent for their daily bread on the gifts of the nursing and domestic staffs.

In September of last year, in response to an urgent appeal in the *Times*, it was decided to collect a penny weekly from each nurse who wished to help our prisoners in Germany. Preliminary notices were sent to the sisters, asking them to contribute, and to gather the pennies. Stout linen bags were made and numbered for the wards; these are given out every Friday morning, and duly returned to the office, where their contents are counted, entered in a book showing the return for each ward, and finally banked. The total is posted in the dining-room on a special notice-board, where particulars *re* the workings of the scheme are displayed, with any postcards of thanks that come from the men. With some 300 nurses on duty one would expect to get £1 5s. weekly, but the collection always exceeds this amount—the average being well over £2. One often sees threepenny and sixpenny bits, shillings, florins, and even half-crowns amongst the grimy coppers, and once the writer had the joy of finding a dirty piece of governmental paper worth 10s.

In October the scheme was extended to the maids, who were asked to give ½d. a week each. They, too, have responded heartily, and their bags often contain silver surprises! Up to date over £105 has been collected, and each week cheques for 23s. are sent to the Royal Scots Associations and the Black Watch Prisoners of War Relief Fund for parcels for two men in each of these regiments. Besides this, donations are sent from time to time to various regimental committees. It is touching to see the stained postcards that come from the prisoners adopted by the hospital, and it is sufficient reward for any trifling sacrifice to read of their gratitude for the food they receive.

It is hoped that the details here given may induce some other hospitals to adopt a similar scheme. All that is required is the good will of the staff, and the services of someone who can spare an hour or so each week to count, bank, and dispatch the money, and to keep the necessary records.—Yours, etc.,

Private Secretary.

